# Kernel Estimation Using Total Variation Guided GAN for Image Super-Resolution

**DOI:** 10.3390/s23073734

**Published:** 2023-04-04

**Authors:** Jongeun Park, Hansol Kim, Moon Gi Kang

**Affiliations:** School of Electrical and Electronic Engineering, Yonsei University, Seoul 03722, Republic of Korea

**Keywords:** kernel estimation, generative adversarial networks, super-resolution, self-similarity, total variation, KernelGAN, structural information

## Abstract

Various super-resolution (SR) kernels in the degradation model deteriorate the performance of the SR algorithms, showing unpleasant artifacts in the output images. Hence, SR kernel estimation has been studied to improve the SR performance in several ways for more than a decade. In particular, a conventional research named KernelGAN has recently been proposed. To estimate the SR kernel from a single image, KernelGAN introduces generative adversarial networks(GANs) that utilize the recurrence of similar structures across scales. Subsequently, an enhanced version of KernelGAN, named E-KernelGAN, was proposed to consider image sharpness and edge thickness. Although it is stable compared to the earlier method, it still encounters challenges in estimating sizable and anisotropic kernels because the structural information of an input image is not sufficiently considered. In this paper, we propose a kernel estimation algorithm called Total Variation Guided KernelGAN (TVG-KernelGAN), which efficiently enables networks to focus on the structural information of an input image. The experimental results show that the proposed algorithm accurately and stably estimates kernels, particularly sizable and anisotropic kernels, both qualitatively and quantitatively. In addition, we compared the results of the non-blind SR methods, using SR kernel estimation techniques. The results indicate that the performance of the SR algorithms was improved using our proposed method.

## 1. Introduction

High-resolution (HR) images are required in various applications, for example, medical or satellite imaging, wherein specific objects must be distinguished or patterns must be recognized. However, the observed images often have low resolution (LR) because of the physical limitation of the small image sensor or the image acquisition environments. Single image super-resolution (SISR) algorithms for recovering HR images from LR images, have been extensively studied for decades. By overcoming the limitations of the observed LR image, the desired information can be exploited, or the hardware cost efficiency can be achieved. The LR image observation model, also referred to as the degradation model, is described as follows [1]:

(1) y=DBMx+n,

where *y* represents the LR image, *x* represents the HR image, DBM is the degradation operation comprising the downsampling matrix *D*, blurring matrix (blurring kernel, SR kernel, point spread function; PSF) *B*, and warping matrix *M* while *n* represents the additive white noise. Image super-resolution (SR) reconstruction is generally a severely ill-posed problem because the information from an LR image is usually insufficient and the blurring matrix *B* is typically unknown.

**Figure 1:**
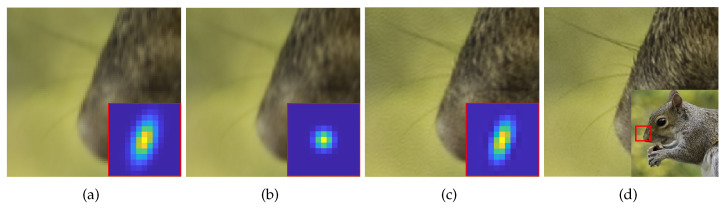
Comparison of SISR results for scale factor of ×2 using [13]. (**a**) input LR image degraded with ground truth(GT) blurring kernel. (**b**) SISR result with a kernel assumed as Gaussian kernel. (**c**) SISR result with an estimated kernel. (**d**) GT image.

To overcome above inherent physical limitations and obtain an accurate HR image, numerous methods have been proposed in two branches: (i) classical approaches [2,3,4,5,6,7,8], and (ii) deep-learning-based approaches [9,10,11,12,13,14,15,16]. In the classical SISR, studies have generally focused on addressing the ill-posedness resulting from insufficient information in the LR image and inaccurate registration by using methods based on regularizing the image prior [2,3,4,5] or exploiting the recurrence property of the internal image patches [6,7,8]. However, the blurring matrix *B* in these methods is usually assumed to be known from measurements or simple blurring such as a Gaussian kernel or bicubic kernel. Early deep-learning-based approaches [9,10,11,12,13,14,15], HR images were degraded using a Gaussian or bicubic kernel to generate LR-HR dataset pairs. However, this dataset generation method is insufficient for representing natural LR images because the blurring matrix *B* varies depending on the image acquisition environment. Because only a single image is given in SISR, the information that can be used for the SR is limited to *B* or the image priors. A comparison between the SISR results using the assumed kernel and the estimated kernel is shown in Figure 1. The SISR result using the assumed kernel in Figure 1b shows a blurry result without any resolution improvement. However, when the estimated kernel is used, the resolution of the SISR result is improved, evident through the clear visibility of whiskers and patterns of fur in Figure 1c. Therefore, the blurring kernels in the SR process have to be considered to improve the performance of the SISR algorithms.

A multitude of methods has been proposed to address this issue in real-world SISR [17,18,19]. Ji et al. [16] proposed a method inspired by KernelGAN [20] that constructs a kernel pool from a high-quality source image using kernel estimation techniques before generating an LR image through degradation. This demonstrates that the degradation process, particularly the blurring process, can be effectively modeled using kernel estimation methods. Despite its advantages, KernelGAN may exhibit inconsistencies or instabilities owing to the inherent randomness of GAN. Liang et al. [21] introduced a kernel-pool generation method, flow-based kernel prior (FKP), which exploits invertible mapping between a random variable and a kernel using several flow blocks. It achieved stable kernel estimation performance. However, their method required pre-training and could not estimate an accurate kernel if the desired kernel was not included in the kernel pool. Kim et al. [22] proposed an enhanced version of KernelGAN that exploits the distinctive properties of LR-HR image pairs. Their method demonstrated improved performance compared to KernelGAN, but still encountered challenges in estimating sizable and anisotropic kernels.

For this study, we proposed a kernel estimation method that addresses the challenge of accurately estimating sizable and anisotropic kernels. The proposed method is guided by a total variation map, which emphasizes the edge regions of the image where detailed information is most prevalent, and exploits self-similarity to a greater extent than previous methods. The main contributions of the study are summarized as follows:

- The proposed method adopts a total variation map and uses it as a guide for the network to focus on the structural information of the image.
- Compared to previous methods, the proposed method is cost- and memory-efficient.
- We demonstrate that the proposed method exhibits superior performance, particularly in accurately estimating sizable and anisotropic kernels, compared to conventional methods.

The remainder of the paper is organized as follows: In Section 2, a summary of the relevant background work is provided. The proposed method is described in detail in Section 3. The experimental results are presented in Section 4, and the conclusions are presented in Section 5.

## 2. Background

As mentioned in the previous section, the blurring matrix *B* is generally assumed to be a Gaussian or a bicubic kernel in various SISR studies. However, owing to environmental factors such as camera shaking, rapid movement, and weather conditions, the blurring kernel may not be identical even if the same imaging system is used. For SISR, accurately estimating the blurring kernel is crucial because an inaccurately assumed kernel often produces reconstructed images with ringing or blurring artifacts.

Michaeli et al. [7] proposed an SR kernel estimation method for a single image using the self-similarity property of natural images, in which similar structures are repeated across scales. In their method, patches with explicit structural similarities were matched, and the SR kernel was estimated using maximum a posteriori (MAP) optimization. KernelGAN, proposed by Bell-Kligler et al. [20], is a pioneering work that introduced a deep linear network for SR kernel estimation. Although having the same fundamental background, it employs a distinct optimization tool, GAN. In KernelGAN, the generator *G* generates a fake patch by downscaling a patch randomly picked from the input image, and the discriminator *D* determines whether it is a fake or real patch of the input image. KernelGAN is trained to create a downscaled fake patch with the same statistics as a real patch, maximizing self-similarity, such that the network reproduces the degradation process of the given input image and extracts the optimal SR kernel. KernelGAN demonstrated that the network could successfully estimate the SR kernels.

Kim et al. [22] noted that KernelGAN did not consider image sharpness and the difference in edge thickness between HR and LR images and proposed Enhanced-KernelGAN (E-KernelGAN). They consider the image’s sharpness using ‘degradation and ranking comparison’, which indirectly utilizes the structural information of the image and improves the kernel estimation stability by excluding unsuitable candidates from the kernel space. In addition, they proposed the ‘kernel correction’ module as a post-processing step to refine the estimated kernel variance and resize it. This post-processing step, which considers edge thickness, also indirectly uses structural information. E-KernelGAN successfully improves the SR kernel estimation stability and accuracy, but fails to fully exploit the self-similarity property that is fundamental to SISR kernel estimation. Consequently, the estimation of sizable and anisotropic SR kernels is limited. In the next section, we propose Total Variation Guided KernelGAN (TVG-KernelGAN), which efficiently utilizes self-similarity by weighting the input image.

## 3. Proposed Method

### 3.1. Challenging Kernels and SR

Classical SR methods typically estimate HR images by solving the optimization problem as follows:

(2) $$\begin{array}{c} \hat{x}=\underset{x}{argmin}\left\{{∥y-DBMx∥}_{2}^{2}+\lambda {∥\nabla x∥}_{p}^{p}\right\}. \end{array}$$

$\hat{x}$ is the optimal HR image that minimizes the given cost function. The first term is the data fidelity term, and the second term is the *p*-norm regularization term, which imports various image priors to suppress noise. λ is the regularization parameter that determines how much the regularization term contributes to the optimization process. In SISR, *M* is not considered because the given image can be located at arbitrary coordinates. *D* is a downsampling matrix the inverse of which is generally interpreted as an arbitrary interpolator, such as a bilinear or bicubic interpolator. The remaining factors that affect the SR performance are *B* and the regularization term. Meanwhile, deep-learning-based SR methods typically use Equation (3) to predict HR images.

(3) $$\begin{array}{c} \hat{x}=F\left[\underset{\Theta }{argmin}\left\{\sum_{i}{∥F\left(\Theta ,y_{i}\right)-x_{i}∥}^{2}\right\},y\right],\\ \mathrm{where},y_{i}=DBMx_{i}+n. \end{array}$$

**Figure 2:**
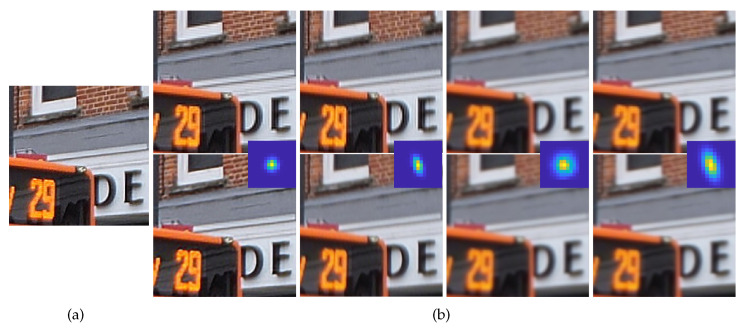
SR results comparison using blurring kernels in scale factor ×2. (**a**) GT, (**b**) the first row shows the results of solving Equation (2) and the second row shows the results of [13]. The input images of each column are degraded, respectively, from GT using the different blurring kernels shown in the middle of each columns. However, in the SR process, both SR methods assumed the same Gaussian kernel as a blurring kernel to investigate the effects of different kernels.

*F* is the SR network output with the network parameter Θ and input image *y*. Data pairs $\left(x_{i},y_{i}\right)$ are prepared using the degradation model in Equation (1). In general, the blurring kernel *B* is assumed to be bicubic or Gaussian, which limits the generalization performance of the network. To investigate the effects of different blurring kernels on the SR results, we degraded the same input image using four differently shaped kernels and applied the SR methods as shown in Figure 2. When the input image was degraded with a small round kernel, the resulting SR image showed relatively weak ringing artifacts and blurring artifacts, as shown in the first column of Figure 2b. The ringing and blurring artifacts became more severe in the second column of Figure 2b with the change to an anisotropic kernel. With a sizable kernel in the third column of Figure 2b, the SR results show severe blurring artifacts without any noticeable resolution enhancement. In the case of a sizable and anisotropic kernel in the fourth column of Figure 2b, severe blurring and ringing artifacts can be found. These results show that more sizable and anisotropic kernels severely deteriorate the SR performance; the focus of this study is this kind of kernel. As shown in Section 4, previous work on kernel estimation failed to estimate these sizable and anisotropic kernels. In this paper, we propose a method that successfully estimates these challenging kernels.

### 3.2. Total Variation Weight Map

When a given input image is severely blurred, the network has difficulty extracting meaningful features from the given patch to distinguish between real and fake patches. Consequently, the network may converge to a meaningless local minimum. To estimate the sizable and anisotropic kernels successfully in such situations, we focused on the edges of the images. Several studies on SR and kernel estimation have used edges that contain rich structural information [15,23,24,25]. In particular, Cho and Lee [24] estimated an extremely directional and sharp kernel, generally known as a motion-blurring kernel, using a strong edge prior. This implies that strong edges that remain after the blurring process are still present when the same blurring process is applied. Inspired by this edge prior, we incorporated the edges of the input image into the kernel estimation process.

The proposed method aims to maximize self-similarity efficiently by weighting the edge region of the input image such that the network can focus on structural information and successfully estimates more challenging SR kernels. Because we are interested in the edge region rather than the edge itself, we require a relatively smooth weight around the edges. Farsiu et al. [2] proposed total variation using four directions, including two diagonal directions, to regularize the noise. They demonstrated that this regularization suppressed noise while preserving the edges, meaning that the total variation smoothly and gradually highlighted the edges and details.

**Figure 3:**
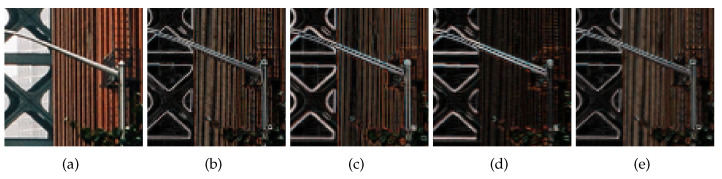
Normalized weights map examples, (**a**) GT, (**b**) Using forward difference for two directions and L2 norm, (**c**) Using Sobel filtering and L1 norm, (**d**) Using forward difference for two directions and L1 norm, (**e**) Using forward difference for four directions and L1 norm.

There are several options for highlighting the edges, as shown in Figure 3. The weight maps were normalized using the maximum values of each map. Consistent with [2], Figure 3e showed the smoothest edge map with the smallest weight difference between the strong and weak edges. Therefore, we used the following four-direction total variation map:

(4) $$\begin{array}{c} {\mathrm{map}}_{TV}=\underset{a+b\geq 0}{\underset{︸}{\sum_{a=0}^{1}\sum_{b=-1}^{1}}}||y-P_{n1}^{a}P_{n2}^{b}y{\left|\right|}_{1},\\ w={\mathrm{map}}_{TV}+c, \end{array}$$

where *P* is the shift operator in the vertical direction $n_{1}$ and horizontal direction $n_{2}$, and *a* and *b* are the order of *P*. We focus on the edge region of the input image; however, this does not mean that the plane region has no information. Therefore, we added a constant *c* to $map_{TV}$ so that the plane region is not completely discarded from the kernel estimation process.

### 3.3. TVG-KernelGAN

**Figure 4:**
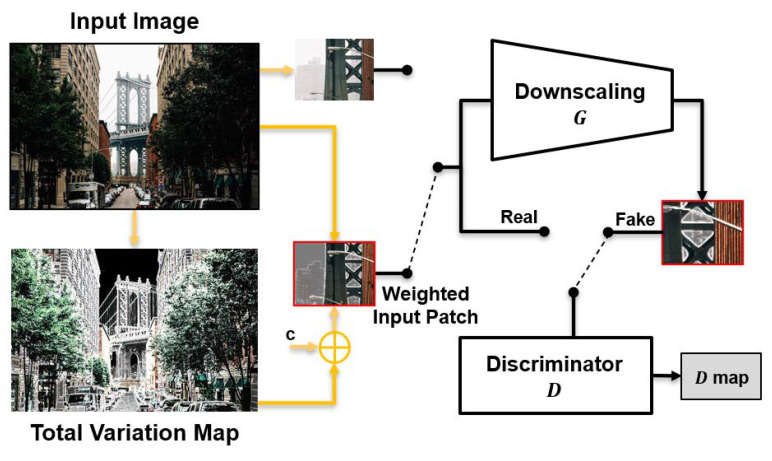
Structure of TVG-KernelGAN.

The structure of the proposed method is shown in Figure 4. The input image is first weighted by the total variation map and used as an input patch for *G* and *D*. By incorporating the total variation map as a weight, the network directly utilizes the structural information of the input image and focuses on the edge region. The weighted input is not used during the entire training process but is instead used at certain iterations, i.e.,

(5) $$\hat{y}=\left\{\begin{array}{cc} w*y, & \mathrm{if}\;\mathrm{mod}(t,s)=0\\ y, & \mathrm{otherwise}, \end{array}\right.$$

where $\hat{y}$ is the input for *G* and *D* at iteration *t*, and *s* is the ratio parameter that determines the frequency of using the weight map *w*. In the same context as the addition of the constant *c* to ${\mathrm{map}}_{TV}$ in Equation (4), this switching scheme ensures that the information in the plane region is not completely discarded. Furthermore, the total variation guide scheme is applied after several tens of iterations when a general bicubic kernel shape is sufficiently formed.

Finally, the TVG-KernelGAN loss function is given by

(6) $$L_{TVG-KernelGAN}=|D\left(\hat{y}\right)-1|+|D\left(G\left(\hat{y}\right)\right)|+R_{B}.$$

Here, $R_{B}$ is the kernel regularization term as in [20], and $R_{B}$ is given as follows:

(7) $$R_{B}=\alpha K_{energy}+\beta K_{boundary}+\gamma K_{sparse}+\delta K_{center}.$$

*K* terms represent the kernel losses that force the kernel extracted from *G* to be meaningful. $K_{energy}$ make the kernel conserve the energy of the input data; $K_{boundary}$ and $K_{sparse}$ make the kernel not be an over-smoothing kernel; and $K_{center}$ centers the kernel. α, β, γ, and δ are the regularization parameters of *K* terms, respectively. Because the total variation guiding scheme requires only simple calculations on the input image and no additional network, the proposed method efficiently improves the kernel estimation performance with less additional cost and memory than KernelGAN and E-KernelGAN.

## 4. Experimental Results

We evaluated our method using three datasets: DIV2KRK, Flickr2KRK and DIV2KSK. The DIV2KRK dataset consists of 100 validation images from DIV2K [26] degraded with random kernels that were generated in [20] to follow an anisotropic Gaussian random distribution and applied by multiplicative noise. Similarly, Flickr2KRK was generated using the first 100 images in Flickr2K [27] by applying the same kernel generation process. In both datasets, we shuffled 100 kernels and used them to degrade and downsample the ground truth (GT) images for scale factors of ×2 and ×4. However, these datasets lack sufficiently sizable and anisotropic kernels, and have meaningless kernels with several isolated peaks. To evaluate the performance of the kernel estimation on sizable and anisotropic kernels, we generated a new dataset named the DIV2KSK (DIV2K Synthetic Kernel). We randomly selected 15 validation images from DIV2K [26], and degraded and downsampled them using 16 synthetic kernels for scale factors of ×2 and ×4, respectively, to produce total 240 input images.

We implemented our algorithm using the Python PyTorch library and trained it using an NVIDIA GeForce RTX 3090 GPU. For training, we set the initial learning rate to $2\times 10^{-4}$ and trained the network for 3000 iterations using the ADAM optimizer with $\beta_{1}=0.5$ and $\beta_{2}=0.999$. The parameters *c* and *s* were set to 0.6 and 2, respectively.

### 4.1. Kernel Estimation Results

We evaluated our method by comparing it with the conventional kernel estimation algorithms; KernelGAN [20], FKP-KernelGAN [21], E-KernelGAN [22] and E-KernelGAN-DIP. The E-KernelGAN-DIP utilizes a deep image prior (DIP) [28] network to estimate more reasonable kernels. To quantitatively evaluate the estimated kernels, we used two metrics: kernel error(KE) and kernel similarity(KS), as follows:

(8) $$\begin{array}{ll} KE & =\;∥B^{GT}-\hat{B}{∥}_{2}^{2},\\ KS & =\frac{B^{GT}\cdot \hat{B}}{∥B^{GT}{∥}_{2}{∥\hat{B}∥}_{2}}. \end{array}$$

KE is the sum of the difference squares between the GT kernel $B^{GT}$ and the estimated kernel $\hat{B}$. KE represents the errors of the values of $\hat{B}$ to those of $B^{GT}$. However, it tends to be low when $\hat{B}$ is a round shape and large enough. To address this limitation, we introduce a metric KS similar to that proposed in [29] to evaluate the shape similarity between $B^{GT}$ and $\hat{B}$. To ensure a fair comparison, all kernels, including the ground truth kernels, are moved for their center of mass to be centered because we do not consider image shift. In addition, to analyze the relationship between the kernel estimation performance of the algorithms and the GT kernel size, we introduce the kernel size *r* as follows:

(9) $$\begin{array}{c} M^{T}=\left\{\begin{array}{cc} 1, & \mathrm{if}B_{i}>T,\\ 0, & \mathrm{otherwise}, \end{array}\right.\mathrm{where}i\in B,T=\frac{\max(B)}{30},\\ r=\frac{\sum_{i}M_{i}^{T}}{N_{1}N_{2}}. \end{array}$$

$M^{T}$ is a binary mask where the elements of *B* greater than the threshold *T* are marked, *i* is the location of the kernel space, and $\left(N_{1},N_{2}\right)$ is the kernel space size, that is, (17,17) in the case of a scale factor ×2. The region marked by $M^{T}$ captures most of the kernel energy of a given kernel (at least 95 percent of the total kernel energy).

**Figure 5:**
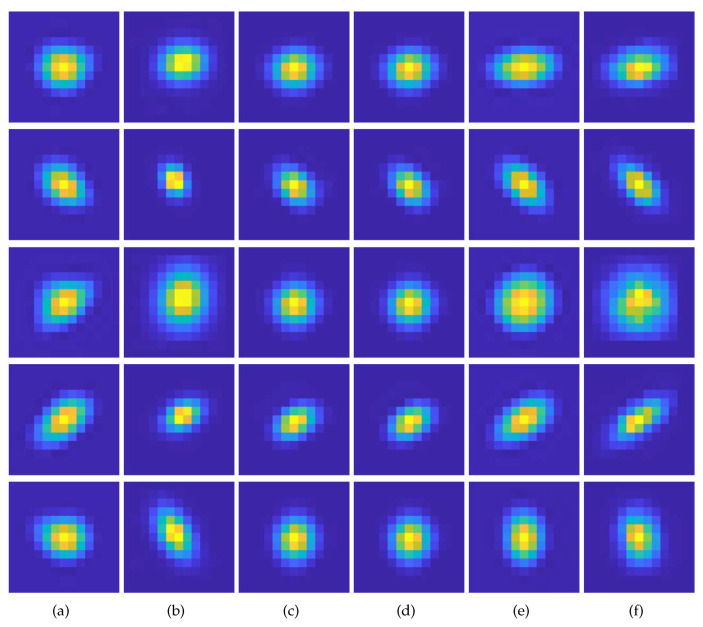
Qualitative results of the kernel estimation for scale factor of ×2 for DIV2KRK dataset. (**a**) KernelGAN [20], (**b**) FKP [21], (**c**) E-KernelGAN [22], (**d**) E-KernelGAN-DIP [22], (**e**) TVG-KernelGAN, (**f**) GT.

**Figure 6:**
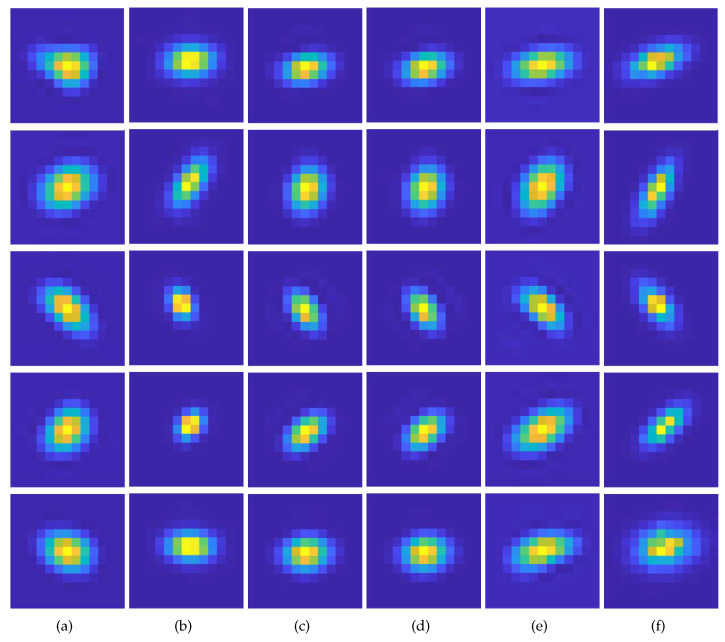
Qualitative results of the kernel estimation for scale factor of ×2 for Flickr2KRK dataset. (**a**) KernelGAN [20], (**b**) FKP [21], (**c**) E-KernelGAN [22], (**d**) E-KernelGAN-DIP [22], (**e**) TVG-KernelGAN, (**f**) GT.

**Figure 7:**
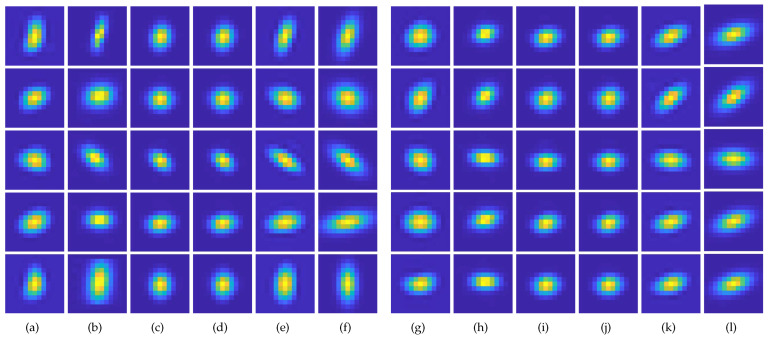
Qualitative results of the kernel estimation for scale factor of ×2 for DIV2KSK dataset. (**a**,**g**) KernelGAN [20], (**b**,**h**) FKP [21], (**c**,**i**) E-KernelGAN [22], (**d**,**j**) E-KernelGAN-DIP [22], (**e**,**k**) TVG-KernelGAN, (**f**,**l**) GT.

First, a qualitative comparison of kernel estimation results on the DIV2KRK and Flickr2KRK datasets for scale factor of ×2 is shown in Figure 5 and Figure 6, respectively. KernelGAN often fails to estimate the direction and overall shape of a kernel, including its length and thickness. FKP-KernelGAN(FKP) attempted to estimate the kernel as closely to the GT kernel as possible, but it had clear limitations, as it could not present kernels on which it had not previously been trained. In the case of E-KernelGAN and E-KernelGAN-DIP, although they could stably estimate the kernel direction, the shape of the estimated kernels tended to be relatively small, round-shaped, and short compared to the GT kernels. Our proposed method, TVG-KernelGAN, estimates kernels that approximate the GT kernels, regarding both the kernel direction and overall shape. However, for small and sharp kernels such as the kernel in the 4th row of Figure 6f, TVG-KernelGAN tended to estimate kernels thicker than the GT kernel. Next, a qualitative comparison of the kernel estimation results on the DIV2KSK dataset for the scale factor of ×2 is shown in Figure 7. The kernel estimation tendencies were similar to those of the two previous two datasets. KernelGAN was unstable and inaccurate, FKP had obvious limitations, and the results of E-KernelGAN and E-KernelGAN-DIP still had insufficient length. By contrast, TVG-KernelGAN outperformed the other conventional methods, as the GT kernels were large and anisotropic.

**Figure 8:**
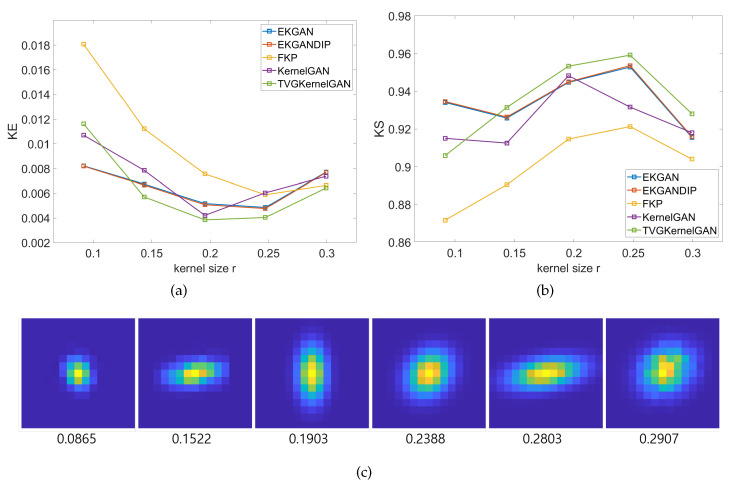
KE and KS curves according to the kernel sizes *r*. (**a**) KE, (**b**) KS, (**c**) the kernel examples and the corresponding kernel sizes *r*.

A quantitative comparison of the kernel estimation results for the entire dataset and for the scale factors of ×2 and ×4 is shown in Table 1. A lower value of KE and a higher value of KS indicate better performance. The numbers in red indicate the first-highest scores. For the DIV2KRK dataset, E-KernelGAN-DIP achieved the highest scores for both KE and KS, followed by E-KernelGAN, which achieved a score almost identical to that of E-KernelGAN-DIP. TVG-KernelGAN achieved the third-highest score. For the Flickr2KRK dataset, TVG-KernelGAN achieved the highest score for KE and the second-highest score for KS, whereas E-KernelGAN-DIP achieved the second-highest score for KE and the highest score for KS. E-KernelGAN achieved the third-highest score for both KE and KS. The E-KernelGAN, E-KernelGAN-DIP, and TVG-KernelGAN scores differ little for the two datasets. However, for the DIV2KSK dataset, TVG-KernelGAN achieved a significantly higher score than the other conventional methods. This quantitative comparison was consistent with the qualitative comparison presented above. In addition, Figure 8a,b, respectively, show the mean of KE and KS of all three dataset samples according to kernel size *r*, and Figure 8c shows examples of kernels of various sizes and the corresponding *r* values. TVG-KernelGAN achieved the highest scores for both KE and KS except for the smallest kernel size. These results suggest that the proposed method is more accurate and stable than conventional methods for estimating sizable and anisotropic kernels.

### 4.2. Non-Blind Super-Resolution Results

In this subsection, we conducted experiments on two branches of SISR, the classical approach and the deep-learning-based approach, to show that the SISR results are improved by using the kernels estimated by the proposed method, particularly for sizable and anisotropic kernels. First, we employed ZSSR [13] as the deep-learning-based approach. Briefly explained, it downscales the input image with a given blurring kernel and then upscales it using a deep-learning upscaling network. It imitates the inverse of the downscaling process for the upscaling network to predict the output SR image after the training session. In this process, the more accurate the blurring kernel is for the downscaling, the higher the SR performance. Next, we employed Equation (2) as a classical approach, by optimizing it using the conjugate gradient descent method. We set $\lambda =1\times 10^{-6}$, p=0.8 and ∇ in the regularization term as a forward difference derivative operator.

**Figure 9:**
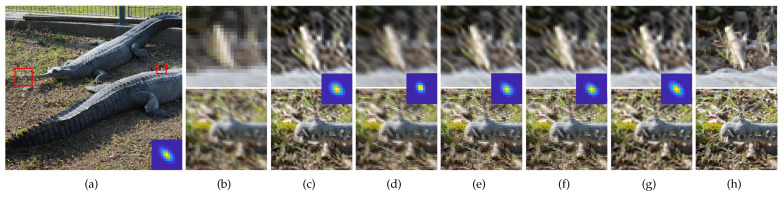
SR results of the 95th image of DIV2KRK dataset using the estimated kernels for scale factor of ×2. The first row is the ZSSR results and the second row is the results of solving Equation (2). From (**c**) to (**g**), the estimated kernels are shown in the middle of each column. (**a**) GT image and kernel, (**b**) LR, (**c**) KernelGAN [20], (**d**) FKP [21], (**e**) E-KernelGAN [22], (**f**) E-KernelGAN-DIP [22], (**g**) TVG-KernelGAN, (**h**) GT.

**Figure 10:**
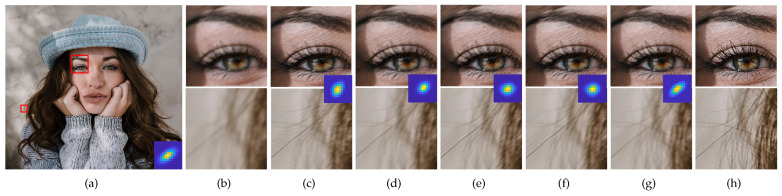
SR results of the 8th image degraded by the 12th kernel of DIV2KSK dataset using the estimated kernels for scale factor of ×2. The first row is the ZSSR results and the second row is the results of solving Equation (2). From (**c**) to (**g**), the estimated kernels are shown in the middle of each column. (**a**) GT image and kernel, (**b**) LR, (**c**) KernelGAN [20], (**d**) FKP [21], (**e**) E-KernelGAN [22], (**f**) E-KernelGAN-DIP [22], (**g**) TVG-KernelGAN, (**h**) GT.

**Figure 11:**
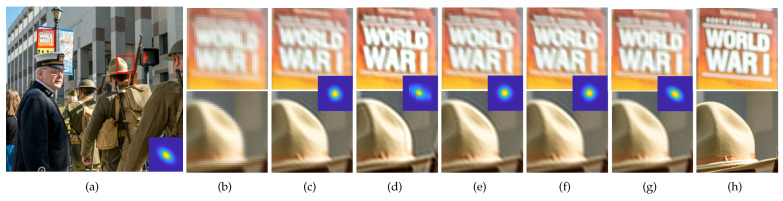
SR results of the 34th image of Flickr2KRK dataset using the estimated kernels for scale factor of ×4. The first row is the ZSSR results and the second row is the results of solving Equation (2). From (**c**) to (**g**), the estimated kernels are shown in the middle of each column. (**a**) GT image and kernel, (**b**) LR, (**c**) KernelGAN [20], (**d**) FKP [21], (**e**) E-KernelGAN [22], (**f**) E-KernelGAN-DIP [22], (**g**) TVG-KernelGAN, (**h**) GT.

**Figure 12:**
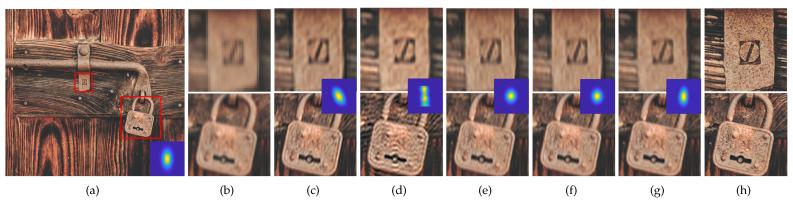
SR results of the 14th image degraded by the 1st kernel of DIV2KSK dataset using the estimated kernels for scale factor of ×4. The first row is the ZSSR results and the second row is the results of solving Equation (2). From (**c**) to (**g**), the estimated kernels are shown in the middle of each column. (**a**) GT image and kernel, (**b**) LR, (**c**) KernelGAN [20], (**d**) FKP [21], (**e**) E-KernelGAN [22], (**f**) E-KernelGAN-DIP [22], (**g**) TVG-KernelGAN, (**h**) GT.

Qualitative comparisons of the SR results obtained using the two SR methods for the scale factor of ×2 are shown in Figure 9 and Figure 10, and that for the scale factor of ×4 are shown in Figure 11 and Figure 12. We observed that wrongly estimated kernels result in artifacts in the SR results. First, when the estimated kernels were small, the SR results exhibited both the ringing and blurring artifacts as shown in Figure 9d and Figure 10d. Second, when the estimated kernels had the incorrect anisotropic direction or round shapes, the SR results exhibited ringing artifacts as shown in Figure 10c,e,f, Figure 11c,e,f and Figure 12c. Lastly, when the estimated kernels had the correct anisotropic direction but insufficient length, the SR results again exhibited ringing artifacts as shown in Figure 9e,f and Figure 10d, or the slightly blurry artifacts as shown in Figure 12e,f. In contrast, the SR results using the kernels estimated by the proposed method showed resolution enhancement with much less or no ringing artifacts as shown in Figure 9g, Figure 10g, Figure 11g and Figure 12g. For a quantitative comparison, we also measured PSNR and SSIM between the GT images and the SR results images for the entire dataset and the scale factor of ×2 as shown in Table 2. E-KernelGAN and E-KernelGAN-DIP achieved superior or similar scores with TVG-KernelGAN for the first two datasets. However, TVG-KernelGAN achieved superior scores for the DIV2KSK dataset with more sizable and anisotropic kernels. These results are consistent with that in Section 4.1, showing that the performance of the SR algorithms using the sizable and anisotropic kernels estimated by the proposed method has been improved.

### 4.3. Memory and Cost Efficiency

We evaluated the cost and memory efficiency of the KernelGAN series, including KernelGAN, E-KernelGAN, E-KernelGAN-DIP, and TVG-KernelGAN, by measuring the parameter numbers and run-time for 3000 iterations at a scale factor of ×2. The results of these measurements are presented in Table 3. First, owing to the use of a large *D* network, E-KernelGAN had parameters that were 2.5 times more than that of KernelGAN, and required several times more run-time for kernel estimation than KernelGAN. Furthermore, as E-KernelGAN-DIP utilizes the DIP network, it has significantly more parameters and requires a much longer time, as shown in Table 3. However, because we did not construct any additional networks, TVG-KernelGAN has the same parameter numbers as KernelGAN and takes same time as KernelGAN, making it much more time-efficient than E-KernelGAN and E-KernelGAN-DIP. These results suggest that the TVG-KernelGAN can efficiently leverage self-similarity in the input image with a simple modification.

### 4.4. Limitation

As shown in Section 4.1 and Section 4.2, TVG-KernelGAN performed better in estimating sizable and anisotropic kernels. However, it achieved lower scores of KE and KS for the smallest kernel size *r* as shown in Figure 8, achieving even lower scores than those of KernelGAN. In a comparison of PSNR and SSIM scores in Table 2, TVG-KernelGAN showed lower scores than those of the E-KernelGAN series, particularly on the DIV2KRK dataset because the DIV2KRK dataset has many small-size kernels compared to the other two datasets. To our knowledge, TVG-KernelGAN fails to estimate the small-size kernels because we emphasized the edge region of the input image rather than the edge itself to estimate sizable and anisotropic kernels. The input image is less blurry when the degradation blurring kernel is small. Then, the small kernel from *G* can easily minimize the GAN loss by utilizing the relatively sharp edge of the original input image. On the contrary, weighting the edge region makes the original edge thicker, preventing *G* from estimating the small kernel. Therefore, we expect that an adaptive algorithm that utilizes the edge weighting scheme according to the degree of smoothness will help solve this problem.

## 5. Conclusions

In this study, we proposed a kernel estimation method for image super-resolution using GAN guided by a total variation map. We simply weighted the input image using its total variation, which includes four directions, to emphasize the edge region, which has prevalent structural information for the network efficiently to maximize the self-similarity of the given input image. The experimental results, including the qualitative and quantitative evaluations, demonstrate that the proposed method estimates the SR kernels more accurately and stably than conventional methods, particularly for sizable and anisotropic kernels. The super-resolution results further show that the proposed method is superior to the compared methods. In addition, the network parameter numbers and run-time measurements demonstrate the efficiency of the proposed method, which simply modifies the input data.

## Figures and Tables

**Table 1:** Comparison of the kernel estimation results in terms of quantitative score, kernel error (KE) and kernel similarity (KS).

			KernelGAN	FKP	E-KernelGAN	E-KernelGAN-DIP	TVG-KernelGAN
DIV2KRK	×2	KE	0.0067	0.0072	0.0043	0.0043	0.0046
		KS	0.9294	0.9239	0.9574	0.9579	0.9543
	×4	KE	0.00088	0.00080	0.00062	0.00062	0.00070
		KS	0.9537	0.9537	0.9698	0.9699	0.9680
Flickr2KRK	×2	KE	0.0087	0.0106	0.0081	0.0080	0.0077
		KS	0.8989	0.8833	0.9094	0.9100	0.9097
	×4	KE	0.00111	0.00093	0.00090	0.00089	0.00089
		KS	0.9391	0.9392	0.9550	0.9550	0.9552
DIV2KSK	×2	KE	0.0051	0.0072	0.0058	0.0057	0.0043
		KS	0.9446	0.9138	0.9426	0.9431	0.9547
	×4	KE	0.00088	0.00100	0.00083	0.00083	0.00074
		KS	0.9478	0.9419	0.9577	0.9579	0.9593

**Table 2:** Comparison of PSNR and SSIM scores of the SR results using estimated kernels.

			Bicubic	KernelGAN	FKP	E-KernelGAN	E-KernelGAN-DIP	TVG-KernelGAN	GT
DIV2KRK	ZSSR	PSNR	28.6953	28.7329	28.3635	29.3803	29.3544	29.0642	29.8799
		SSIM	0.8035	0.8360	0.8413	0.8472	0.8470	0.8416	0.8656
	Equation (2)	PSNR	28.6953	30.0237	28.9431	30.3637	30.3741	30.3425	31.5232
		SSIM	0.8035	0.8516	0.8329	0.8573	0.8576	0.8562	0.8801
Flickr2KRK	ZSSR	PSNR	28.0653	28.4859	27.5576	28.7836	28.7809	28.5700	29.4258
		SSIM	0.7897	0.8230	0.8281	0.8297	0.8296	0.8281	0.8500
	Equation (2)	PSNR	28.0653	29.2672	28.5526	29.3507	29.3542	29.2909	29.0367
		SSIM	0.7897	0.8385	0.8222	0.8404	0.8406	0.8406	0.8341
DIV2KSK	ZSSR	PSNR	24.5548	25.3626	24.7414	25.4244	25.4294	25.4313	26.2298
		SSIM	0.6874	0.7507	0.7514	0.7499	0.7496	0.7529	0.7921
	Equation (2)	PSNR	24.5548	25.9113	25.1470	25.7892	25.7525	25.9618	26.8826
		SSIM	0.6874	0.7608	0.7300	0.7566	0.7563	0.7629	0.7975

**Table 3:** Run-time for scale factor of ×2 and the network parameters of KernelGAN series.

	KernelGAN	E-KernelGAN	E-KernelGAN-DIP	TVG-KernelGAN
Network parameters	181 k	464 k	2824 k	181 k
Run-time	57 s	356 s	930 s	57 s

## Data Availability

Not applicable.
